# Target volume delineation in breast conserving radiotherapy: are co-registered CT and MR images of added value?

**DOI:** 10.1186/1748-717X-9-65

**Published:** 2014-02-26

**Authors:** Mirjam Mast, Emile Coerkamp, Mark Heijenbrok, Astrid Scholten, Wim Jansen, Erik Kouwenhoven, Jasper Nijkamp, Stephanie de Waard, Anna Petoukhova, Henk Struikmans

**Affiliations:** 1 Radiotherapy Centre West, Lijnbaan 32, The Hague, 2501 CK, The Netherlands; 2 Department of radiology, Medical Center Haaglanden, Lijnbaan 32, The Hague, 2501 CK, The Netherlands; 3 Department of clinical oncology, Leiden University Medical Centre, Albinusdreef 2, Leiden, 2300 RC, The Netherlands; 4 Department of Radiation Oncology, Netherlands Cancer Institute – Antoni van Leeuwenhoek Hospital, Plesmanlaan 121, Amsterdam, 1066 CX, The Netherlands

**Keywords:** Breast cancer, CT, MRI, Radiotherapy, Target volume delineation

## Abstract

**Introduction:**

In breast conserving radiotherapy differences of target volume delineations between observers do occur. We evaluated whether delineations based on co-registered computed tomography (CT) and magnetic resonance (MR) imaging may result in an improved consistency between observers. We used the delineation conformity index (CI) to compare clinical target volumes of glandular breast tissue (CTV breast) and lumpectomy cavity (LC) on both imaging modalities.

**Methods and materials:**

Four observers delineated CTV breast and LC on co-registered CTMR images in ten breast cancer patients. CIs were determined for CT and CTMR. Furthermore, the Cavity Visualization Score (CVS) of LC was taken into account.

**Results:**

The mean CI for CTV breast (CI_CT;CTV_: 0.82 and CI_CT-CTMR;CTV_: 0.80) and LC (CI_CT;LC_: 0.52 and CI_CT-CTMR;LC_: 0.48) did not differ significantly (p = 0.07 and p = 0.33, respectively). Taking CVS into account for the LC, with a CVS ≥ 4 the mean CI was 0.62 for both CI_CT;LC_ and CI_CT-CTMR;LC_.

**Conclusion:**

The mean volume of the delineated glandular breast tissue based on CT was significantly larger compared to the volume based on CTMR. For patients with a CVS ≥ 4, the mean CIs of the LC were higher compared to CVS < 4 for volumes delineated on both CT as well as CTMR images. In our study cohort no significant differences between the CIs of the CTV breast and the LC delineated on CTMR co-registered images were found compared to the CIs on CT images only. Adding MR images does not seem to improve consistency of the delineation of the CTV breast and the LC, even though the volumes were copied from CT images. Since we included only ten patients, caution should be taken with regard to the results of our study.

## Background

There can be substantial differences in identification of the target volumes among radiation oncologists specialized in breast cancer radiotherapy [1]; even when written delineation guidelines are used [2-4]. Compared to computed tomography (CT) magnetic resonance imaging (MRI) may reveal more relevant details [5]. And, according to Jolicoeur et al., the use of MRI improved the level of agreement between observers delineating the lumpectomy cavity compared to CT [6]. In our former study, published in 2011, we noted that the concordance for delineation of the volumes on CT differed only slightly from the concordance based on magnetic resonance (MR) images [7]. Whether the use of a co-registration of the two imaging modalities could lead to an improvement of the agreement between observers remained unclear.

Therefore, we analyzed the delineation conformity, when based on CT as well as on CTMR co-registered images. In our study, we have evaluated the delineated clinical target volumes of the glandular breast tissue (CTV breast) and the lumpectomy cavity (LC) in ten patients referred for radiation therapy after breast conserving surgery.

## Methods

Between July 2007 and August 2008, fifteen patients with early stage breast cancer (clinically T1-2; N0-1) and treated with breast conserving surgery were included in our study. The mean age was 57 years; 8 patients had right sided and 7 patients had left-sided breast cancer; the tumor was mostly situated in the upper outer quadrant of the breast. Patient and tumor characteristics were described in detail earlier [7]. Since the rigid co-registration was performed on breast markers which were used only in patients 6–15, we included only these ten patients in the present study [8]. After referral for whole breast radiotherapy, a planning-CT scan and directly afterwards a MRI scan were performed, both in supine treatment position. The procedure was described in detail by Giezen et al. [7].

The study was approved by the regional institutional review board METC Zuidwest Holland. All patients agreed to participate in our study by signing an informed consent.

Four observers, i.e. two radiation oncologists and two radiologists, participated in the study and delineated the glandular breast tissue (CTV breast) [7] as well as the lumpectomy cavity (LC) [9]. The four observers delineated CTV breast and LC according to the determined delineation instructions, Table 1[9].

**Table 1:** Delineation instructions for CTV Breast and the lumpectomy cavity

	**CTV Breast**	**Lumpectomy cavity**
Window/Level (WL); Window Width (WW)	- Fixed: WL 0 Hounsfield Unit (HU) and WW of 500 HU for CT and variable WL and WW for MRI.	- Fixed: WL 0 Hounsfield Unit (HU) and WW of 500 HU for CT and variable WL and WW for MRI.
	- Change of WL and WW during delineation permitted for CT and MRI.	- Change of WL and WW during delineation permitted for CT and MRI.
Appearance	- The location of the marking wire, positioned around the palpable Glandular Breast Tissue (GBT), will be used as an aid for CTV Breast delineation.	Appearance of contralateral breast (comparing with ipsilateral breast) serves as aid for LC delineation.
	- The clinical target volume (CTV) breast was defined to comprise all GBT including fatty (involuted) lobes.	
	- Margin of the GBT is (ventrally) assumed to be situated 5 mm below the skin surface; in case of MRI the visible GBT fat is (ventrally) delineated as GBT margin.	
	- Delineation is performed on all CT or MRI slices that are judged to contain GBT.	
	- Appearance of the contralateral breast (by comparing with the ipsilateral breast) on CT or MR images.	
	- The preoperative mammographies and location of the palpable GBT marking wire, visible on CT or MRI, all will serve as an aid for GBT delineation.	
Clips	Surgical clips (if applicable) should all be included within the delineated GBT.	All surgical clips (if applicable) should be included within delineated LC.
Seroma	Postoperative seroma/hematoma present in LC should be included within delineated GBT.	Postoperative seroma/hematoma present in LC should be included within delineated LC.

*Abbreviations:LC* lumpectomy cavity, *WL* window level, *WW* window width, *HU* Hounsfield unit, *MRI* magnetic resonance imaging, *CT* computed tomography.

For all ten patients this resulted in the, for each observer, delineated CTV breast and LC, based on CT images only. After ten weeks, the observers re-evaluated these CTV breast and LC delineations copied on the co-registered CTMR images, and made adaptations when judged necessary. By choosing an interval time of ten weeks it was likely that the observers had forgotten specific details of their CT-based delineations of each specific case. By doing so a more reliable comparison (and eventually an adaptation) between the CT based images and the CTMR images may be achieved. The alternative method of delineating the co-registered CTMR images was not used because this would imply an intraobserver variability.

After defining all CTV breast volumes, a scripting tool was applied to trim all CTV breast volumes up to 5 mm below the skin surface.

To quantify the variability of one delineation compared to another we used the Conformity Index (CI). A CI of 0 indicates no overlap is present between delineations; a CI of 1 indicates completely identical delineations. A method for calculating the CI was used, that is unbiased by the number of observers delineating a target volume [10]. We determined two types of CI of the CTV breast and LC enabling us to assess the influence of imaging modality on delineation variability, and the inter-observer variation, respectively. Firstly, for each observer, the delineated volumes on CT were compared to CTMR, indicated with the symbols CI_CT-CTMR;CTV_ and CI_CT-CTMR;LC_. The resulting CIs were thereafter averaged over the patient population. Secondly, for every delineated target volume we determined the CIs for CT based and CTMR based delineations separately, by comparing the delineations of the different observers to each other. The resulting values are indicated with the symbols CI_CT;CTV_, CI _CTMR;CTV_, CI_CT;LC_ and CI_CTMR;LC_. Again, an average over the patient population was calculated. Furthermore, the earlier assessed “Cavity Visualization Score” (CVS) [9] of the lumpectomy cavity was taken into account in the analysis as well. With the CVS according to Smitt et al. [11] depiction of the lumpectomy cavity is categorized from 1, cavity not visualized, to 5, all cavity margins clearly defined. Finally, a median 3D surface of the CTV breast and LC of all four observers was calculated [12] (local surface variation) in order to analyze and visualize the local interobserver variation for each patient.

### Statistical analysis

Wilcoxon Signed Rank Test was performed to compare all data, CT versus CTMR, since the number of eligible data was less than 30. For analysis we used SPSS Statistics version 17.0. The level of statistical significance was considered p < 0.05 (two sided) for all tests.

## Results

### Glandular breast tissue (CTV breast)

#### ***Delineated volumes***

The mean volume of the delineated glandular breast tissue based on CT (mean 576 cc; range 303–900) was significantly larger compared to the volume based on CTMR (mean 557 cc; range 287–892) (p < 0.01).

#### ***CT versus CTMR: conformity indices and local surface variation***

On the CTMR images few adaptations to the delineated volume were carried out. The range in CIs (CI_CT-CTMR;CTV_) for each observer was 0.89 – 1.00 (mean SD 0.03), Table 2. The mean CI for all observers between CI_CT;CTV_ and CI_CTMR;CTV_ did not differ significantly, Table 3.

**Table 2:** Conformity indices of the CTV breast and lumpectomy cavity (LC) delineations for each observer, CT compared to CTMR

	**CI**_**CT-CTMR, CTV **_**(SD)**	**CI**_**CT-CTMR, LC **_**(SD)**
Observer_1	0.99 (0.01)	0.84 (0.09)
Observer_2	0.89 (0.05)	0.70 (0.23)
Observer_3	1.00 (0.00)	0.91 (0.17)
Observer_4	0.97 (0.05)	0.85 (0.30)

**Table 3:** **Conformity indices (CI**_**CT**_**; CI**_**CTMR**_**) of the CTV breast and lumpectomy cavity (LC) delineations based on CT and CTMR for all observers**

	**CTV breast; mean CI_All (SD)**	**LC; mean CI_All (SD)**
CI_CT_	0.82 (0.04)	0.52 (0.20)
CI_CTMR_	0.80 (0.06)	0.48 (0.21)
p-value CI_CT-CTMR_	0.07	0.33

**Figure 1:**
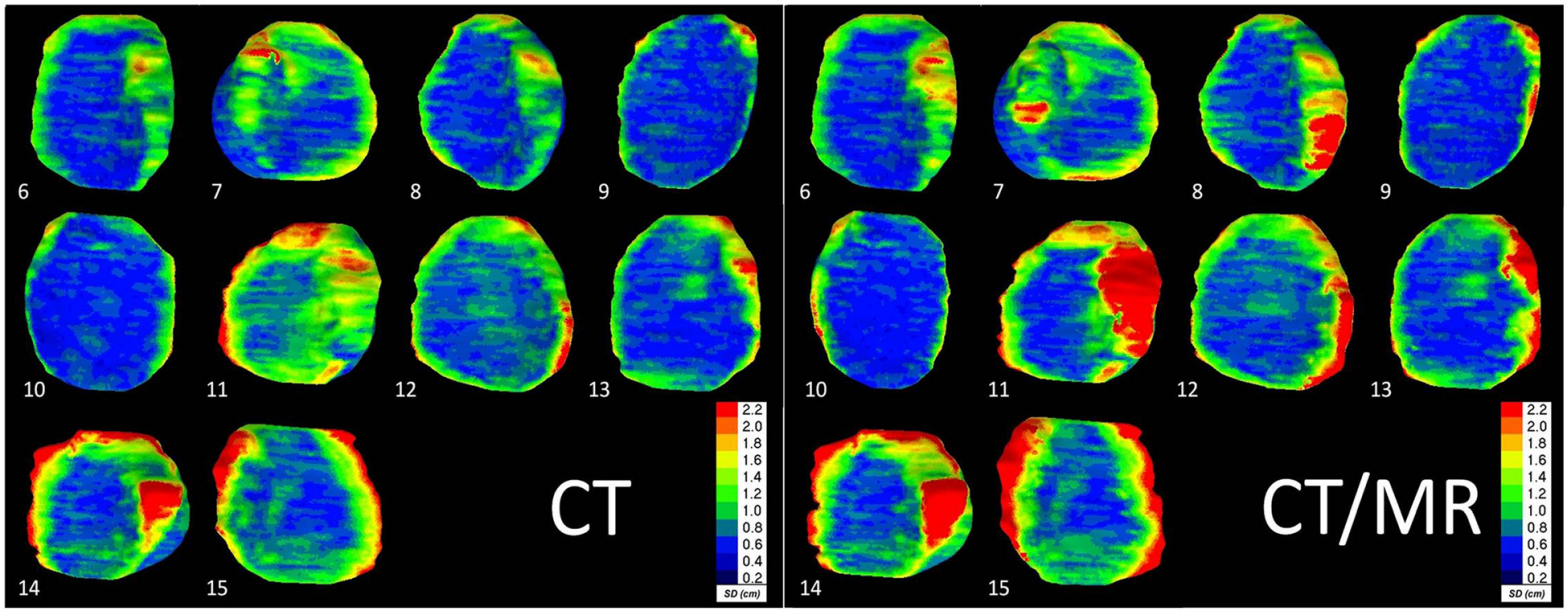
**Left: Coronal posterior view of the ten delineated Clinical Target Volume (CTV) breast Computed Tomography (CT) volumes.** Right: Coronal view of the ten delineated CTV breast CTMR volumes. The local surface distance variation of the four observers is projected on the median surface of each CTV breast. Colour map: Blue: high agreement between observers; Red: low agreement between observers according to the scale given.

The local surface variation in Figure 1 shows again that few adaptations were carried out on the co-registered CTMR images. We found a mean local standard variation between observers of 2.2 mm and 2.6 mm for CT and CTMR, respectively (p = 0.05). In seven out of ten patients the local standard variation increased on CTMR. For patient 8, 11, 12, 13 and 15 the differences were mostly present in the medial part of the CTV breast.

#### ***Interobserver variability***

In considering the variation in the local surface distance, it became apparent that the delineations of the observers varied, on CT as well as CTMR, predominantly in the medial and lateral part of the CTV breast, Figure 1.

### Lumpectomy cavity (LC)

#### ***Delineated volumes***

The mean volumes of the delineated LC based on CT (mean 24 cc; range 4–73) did not differ (p = 0.2) compared to those based on CTMR (mean 26 cc; range 7–71), Table 4.

**Table 4:** Mean volumes for all observers of the lumpectomy cavity (LC) delineations based on CT and CTMR

**Table**4**.**	**Mean lumpectomy cavity volume CT**	**Mean lumpectomy cavity volume CTMR**
Patient_6	28	25
Patient_7	73	71
Patient_8	14	13
Patient_9	7	7
Patient_10	11	11
Patient_11	29	32
Patient_12	4	9
Patient_13	30	34
Patient_14	8	13
Patient_15	33	45

#### ***CT versus CTMR: conformity indices and local surface variation***

For LC more adaptations were carried out than for CTV breast, since the range in CIs (CI_CT-CTMR;LC_) for each observer decreased: 0.70 – 0.91 (mean SD 0.20), Table 2. The mean CI for all observers between CI_CT;LC_ and CI_CTMR;LC_, however, did not differ significantly, Table 3.

**Figure 2:**
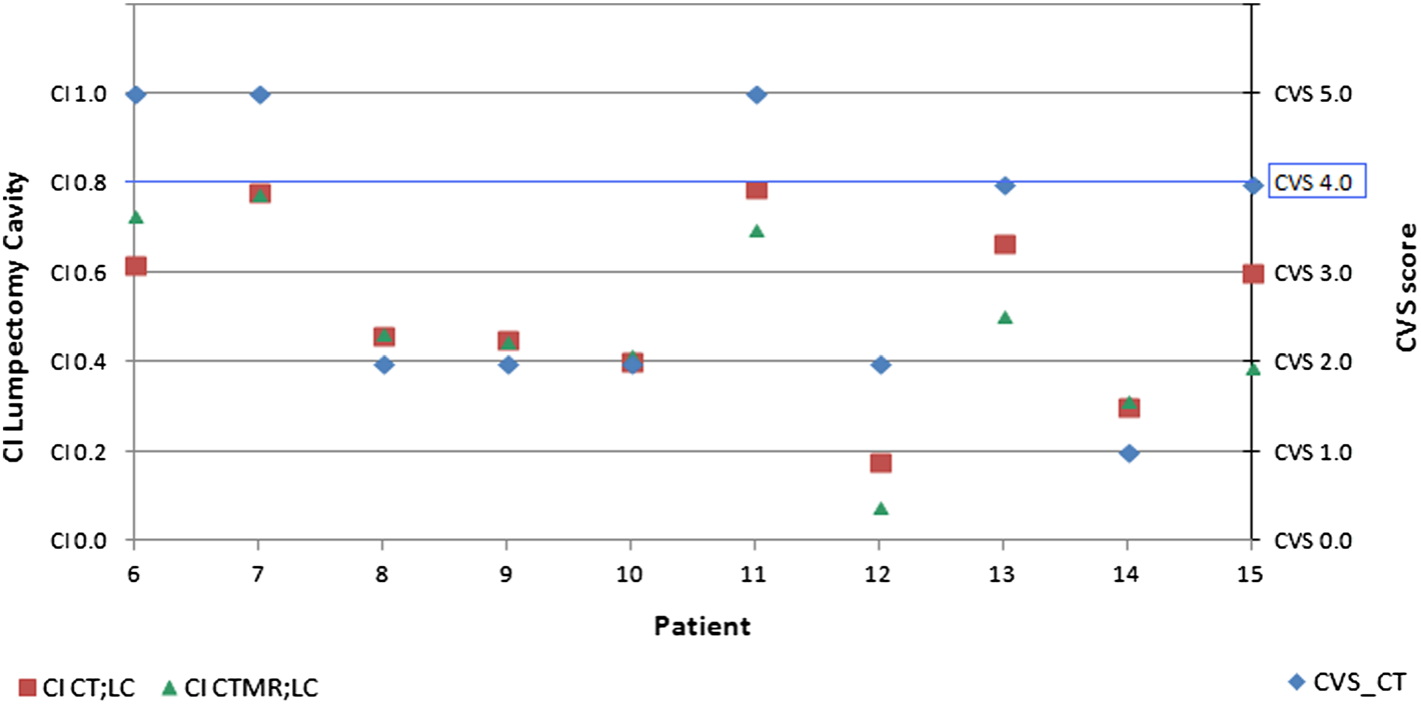
For each patient for the lumpectomy cavity the Conformity Index (CI) on CT, the CI on CTMR and the Cavity Visualization Score (CVS) were determined.

**Figure 3:**
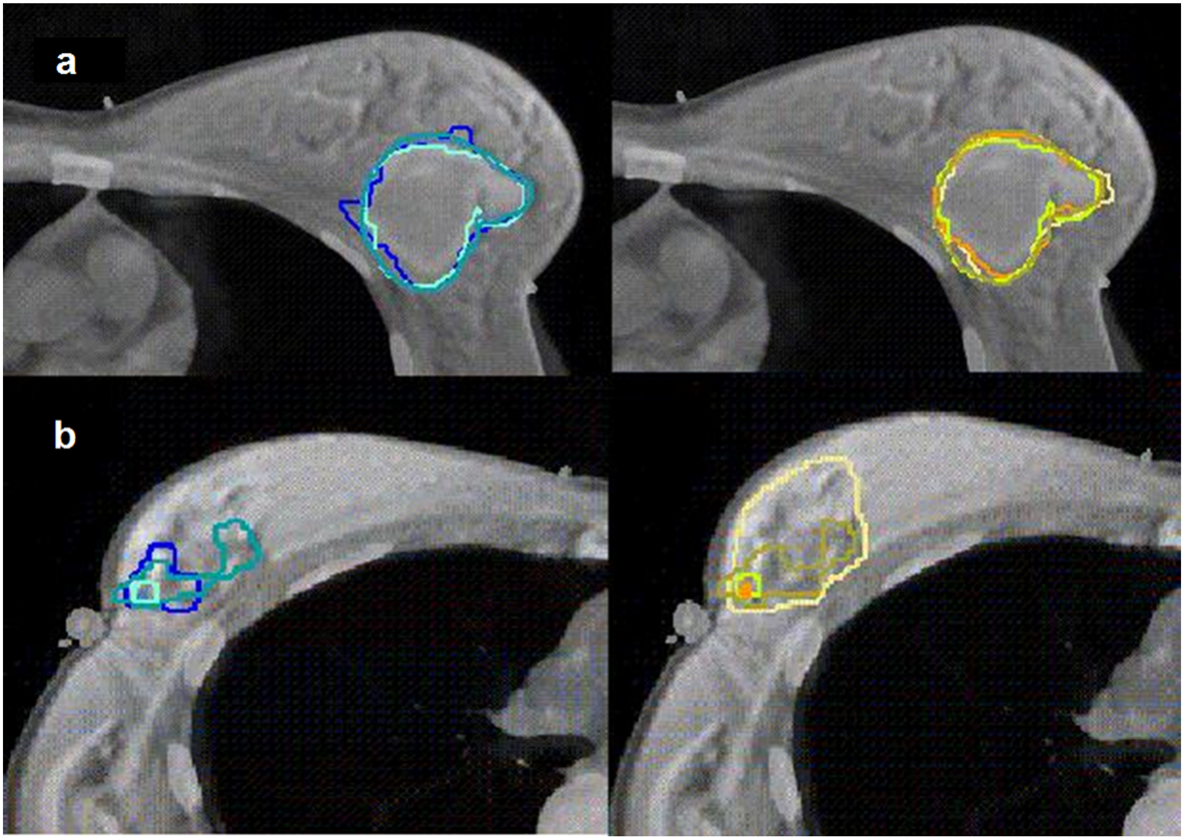
**Differences between the 4 observers on co-registered CTMR images; Left: volumes delineated on CT.** Right: volumes delineated on CTMR; **a**. Example of a patient with seroma, a CVS of 5; **b**. Example of a patient with a CVS of 2.

When taking the CVS into account, we found that, if the CVS was ≥ 4, the mean CI appeared to increase. An increase of the CI to 0.62 was found for CI_CT;LC_ as well as for CI_CTMR;LC_ delineations in all 5 cases with a CVS of ≥ 4. In Figure 2 we display the mean CI of both CT and CTMR on the CVS scale from 0 to 5; see Figure 3 as well.

**Figure 4:**
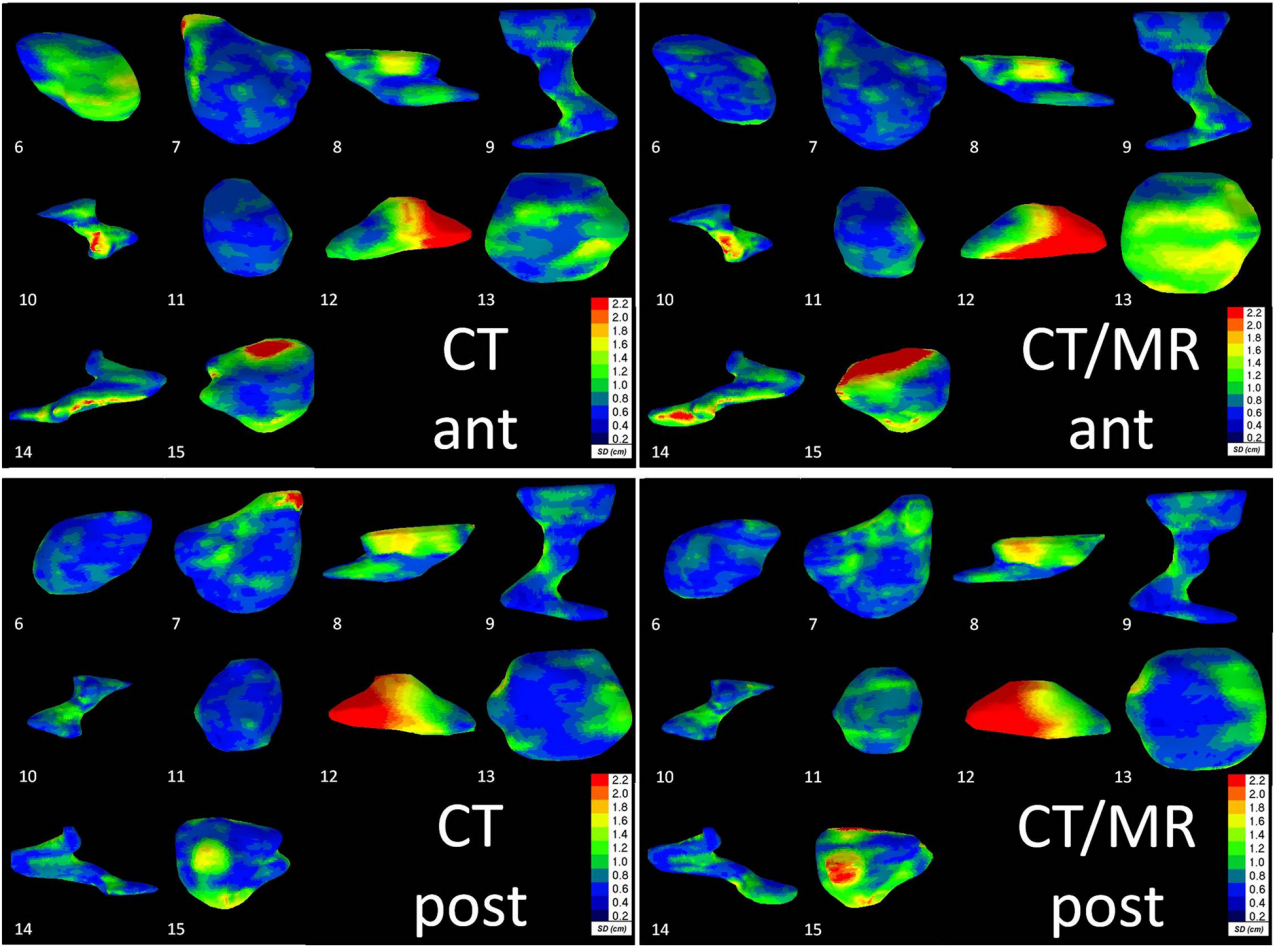
**Left: Coronal view of the ten delineated Lumpectomy Cavity (LC) CT volumes in both anterior as well as posterior view.** Right: Coronal view of the ten delineated LC CTMR volumes in both anterior as well as posterior view. The local surface distance variation of the four observers is projected on the median surface of each LC. Colour map: Blue: high agreement between observers; Red: low agreement between observers according to the scale given.

The local surface distance variation showed more variation in the delineation of the LC compared to CTV breast. We found a mean local standard variation between observers of 2.4 mm and 2.8 mm for CT and CTMR, respectively (p = 0.13). In five out of ten patients (patient 11, 12, 13, 14 and 15) the degree of variability increased on the co-registered CTMR images and in two patients (patient 6 and 7) the degree of variability was larger on the CT images. For the other two patients, no major variability was noted. As an example, in patient 12, a premenopausal patient, no seroma was found, no clips were placed and the CVS was 2, Figure 4.

## Discussion

### CT versus CTMR

In this study we investigated the potential merits of CTMR co-registration on the delineation of the CTV breast and the Lumpectomy Cavity (LC). Concerning the study outline, we only focused on the advantages of CTMR co-registration. Therefore, to avoid intraobserver variability, we copied the CTV breast and LC delineated on the CT to the co-registered CTMR images. Thereafter each observer considered to adapt (yes or no) the CTV breast or LC, respectively when based on the CTMR images. Finally, the differences between the CT based and CTMR based delineations were analyzed. This method could have introduced a bias, since the observers did not delineate the CTMR co-registered images. Comparisons and eventually adaptations were, after an interval time of 10 weeks, done directly on the CTMR co-registered images. In doing so the observers could have been distracted by the copied volume. But the alternative method of delineating the co-registered CTMR images had the disadvantage that this would result in an intraobserver variability between the CT based and the CTMR based delineations.

We found that the CT based CTV breast volumes, when compared with CTMR based volumes, were significantly larger. In our study cohort, it became apparent that the CIs for CTMR co-registered images, when compared to those based on CT images only, did not differ significantly from those based on CT images only, neither for CTV breast nor for LC. With respect to LC, in the 5 cases with a CVS ≥ than 4, the mean CI values increased to 0.62, whereas for the cases with a CVS < 4 a mean CI of 0.50 was found. Compared to the results of our first investigation [9] the CI for the LC increases from 0.32 for MR to 0.48 for the co-registered CTMR.

Remarkably, we found higher CIs (Lumpectomy Cavity) for both CT and CTMR compared to the results of Boersma et al. although our volumes were smaller [4] and in our study the lumpectomy cavity was defined instead of the CTV boost. The CTV boost in the study of Boersma et al. was defined as the 1.5 cm rim of tissue that had surrounded the primary tumor. Also, manual adaptation of the co-registration by each observer could be a reason for the lower CI in the study of Boersma et al., since this could be a bias in the analysis of the delineated structures. In our study, the co-registration was locked after performing the co-registration. Furthermore, in our study clips were placed directly in several segments in the lumpectomy cavity wall representing the extensions of the primary tumor, whereas in the Boersma study clips only had been placed at the deepest (dorsal) border of the lumpectomy cavity [4].

### CTV breast

The major differences in delineation of the target volume between observers were located in the medial and lateral part of the CTV breast. This was confirmed in the study of Li et al. In their study, the effect of these variations on the dose in the organs at risk was studied as well. They concluded, that variations in normal structure dose were found and that large variations in the medial-lateral borders contributed mostly to the variation in the normal structure dose [13]. Therefore, consistency in delineation of the CTV breast is of great importance. In our study cohort specific guidelines (Table 1) were used and consensus meetings had taken place. The latter could explain the non-significant differences in the CTV breast when MR imaging was added.

### Lumpectomy cavity

Delineations of the lumpectomy cavity were done by experienced radiation oncologists and trained radiologists. They used written delineation guidelines (Table 1). All this was in line with the findings of various recent studies. As Wong et al. showed in their study cohort, “trained” oncologists consistently produced smaller target volumes in seroma contouring compared to an “untrained” cohort. The implementation of guidelines reduced the interobserver variability in volume delineation in their study. These data indicated that improved consistency among radiation oncologists may be achieved by consensus guidelines [14].

Furthermore, our results reveal that, when the CVS was ≥ 4, the CI was increased for both CT as well as CTMR defined volumes. This finding was reported before by Landis et al. [1]. This could indicate that, for lumpectomy cavities with a CVS of < 4, specific landmarks such as surgical clips or gold markers may enable a more precise defined CTV boost [3,15]. According to Topolnjak et al. and Park et al., the position of these clips and markers remain stable throughout the treatment course [16,17]. Nevertheless, it seems important to be aware of interfractional target deformations as reported by Ahunbay et al. [18]. Concerning the use of surgical clips, Jolicoeur et al. did not use clips and found a concordance ratio of 0.66 on CT and 0.96 on MR [6].

Finally, as Van Mourik et al. also suggested [3], we confirm that a multi-disciplinary approach is what should be aimed at in target delineation; especially in the delineation of the LC and when the CVS is lower than 4, since every specialist can contribute to a better understanding. If an inconsistency of the surgical clips and at the edge of the seroma was found, as described by Yang et al. [19], this should be part of the multi-disciplinary discussion.

## Conclusion

The mean volume of the delineated glandular breast tissue based on CT was significantly larger compared to the volume based on CTMR. For patients with a CVS ≥ 4, the mean CIs of the LC were higher compared to CVS < 4 for volumes delineated on both CT as well as CTMR images. In our study cohort no significant differences between the CIs of the CTV breast and the LC delineated on CTMR co-registered images were found compared to the CIs on CT images only. Adding MR images does not seem to improve consistency of the delineation of the CTV breast and the LC, even though the volumes were copied from CT images. Since we included only ten patients, caution should be taken with regard to the results of our study.

## Competing interests

The authors have nothing to disclose and indicate no potential conflicts of interests.

## Authors’ contributions

MM: designed, coordinated and performed the study; processed the CT and MR images; collected and analyzed the data; drafted the manuscript. EC, MH, SW, AS: participated in the design of the study; performed the delineation of the target volumes on the CT, MR and CTMR-registered images; helped to draft the manuscript. WJ, EK: participated in the design of the study; helped in collecting the data en performing the analysis; helped to draft the manuscript. HS: designed and performed the study; performed the delineation of the target volumes on the CT, MR and CTMR-registered images; helped to draft the manuscript. AP: designed and performed the study; helped processing the CT and MR images; registered MR to CT images; helped in collecting the data en performing the analysis; helped to draft the manuscript. JN: performed the local surface analysis; helped to draft the manuscript. All authors read and approved the final manuscript.
